# Social determinants of maternal health: a scoping review of factors influencing maternal mortality and maternal health service use in India

**DOI:** 10.1186/s40985-020-00125-6

**Published:** 2020-06-02

**Authors:** Mukesh Hamal, Marjolein Dieleman, Vincent De Brouwere, Tjard de Cock Buning

**Affiliations:** 1 Athena Institute for Research on Innovation and Communication in Health and Life Sciences, VU University, De Boelelaan 1085, 1081 HV Amsterdam, The Netherlands; 2 Maternal and Reproductive Health, Department of Public Health, Institute of Tropical Medicine, Antwerp, Belgium; 3 ISGlobal, Barcelona Centre for International Health Research (CRESIB), Hospital Clínic-Universitat de Barcelona, Barcelona, Spain; 4 KIT Health, PO Box 95001, 1090 HA Amsterdam, The Netherlands

**Keywords:** Social determinants, Structural factors, Intermediary factors, Maternal health, India

## Abstract

**Background:**

Maternal health remains a major public health problem in India, with large inter- and intra-state inequities in maternal health service use and maternal deaths. The Commission on Social Determinants of Health provides a framework to identify *structural* and *intermediary* factors of health inequities, including maternal health, and understand their mechanism of influence, which might be important in addressing maternal health inequities in India. Our review aims to map and summarize the evidence on social determinants influencing maternal health in India and understand their mechanisms of influence by using a maternal health-specific social determinants framework.

**Methods:**

A scoping review was conducted of peer-reviewed journal articles in two databases (PubMed and Science Direct) on quantitative and qualitative studies conducted in India after 2000. We also searched for articles in a search engine (Google Scholar). Forty-one studies that met the study objectives were included: 25 identified through databases and search engines and 16 through reference check.

**Results:**

Economic status, caste/ethnicity, education, gender, religion, and culture were the most important *structural* factors of maternal health service use and maternal mortality in India. Place of residence, maternal age at childbirth, parity and women’s exposure to mass media, and maternal health messages were the major *intermediary* factors. The structural factors influenced the intermediary factors (either independently or in association with other factors) that contributed to the use of maternal health service or caused maternal deaths. The health system emerged as a crucial and independent intermediary factor of influence on maternal health in India. Issues of power were observed in broader social contexts and in the relationships of health workers which led to differential access to maternal healthcare for women from different socioeconomic groups.

**Conclusion:**

The model integrates existing information from quantitative and qualitative studies and provides a more comprehensive picture of structural and intermediary factors of maternal health service use and maternal mortality in India and their mechanisms of influence. Given the limitations of this study, we indicate the areas for further research pertaining to the framework and maternal health.

## Background

Although maternal deaths have declined at the global level, they remain high in many low- and middle-income countries (LMICs), including India, where maternal health continues to be a major public health issue. Maternal health includes women’s health during pregnancy, childbirth, and the post-partum period [1].

India accounted for about one-fifth of the global maternal deaths in 2015 [2], and there are large inter-state and intra-state disparities. Northern states like Assam, Uttar Pradesh (including Uttarakhand), and Rajasthan have a relatively high maternal mortality ratio (MMR) (328, 292, and 255 maternal deaths per 100,000 live births, respectively) compared to southern states like Kerala (66) and Tamil Nadu (90) [3]. Large disparities are also seen in the utilization of maternal health service (antenatal and maternity care) between the states and various population groups within the states, especially among the populations created by socioeconomic divisions [4, 5]. For example, the percentage of institutional delivery is high among women from states like Kerala (99.4%) and Puducherry (99.0%), while it is low among states like Jharkhand (17.7%), Chhattisgarh (18.0%), and Meghalaya and Uttar Pradesh (24.5%) [5]. Inequalities in the utilization of antenatal care (ANC) and skilled birth attendance were observed not only between three studied states (Tamil Nadu, Maharashtra, and Uttar Pradesh) between 1992 and 2006, but also between different economic groups within these states, particularly disadvantaging rural and poor mothers [6]. Such disparities demonstrate maternal health *inequities* in India. Health inequities are disparities in health in access to health service or in health outcomes which are judged to be avoidable, unfair, and unjust [7].

Health inequities are caused by a range of determinants that can be categorized as *structural* and *intermediary determinants* (terms equivalent to *distal* and *proximal* causation, respectively, in sociological literature), according to the Commission on Social Determinants of Health (CSDH) [8]*.* Together, they are termed *social determinants of health*. The *structural determinants* are particularly those that produce health inequities by generating or reinforcing social stratification in societies [8]. They broadly include *socioeconomic and political contexts*, *structural mechanisms*, and *socioeconomic position*. The *socioeconomic and political contexts* include all socioeconomic and political structures, institutions, and relations in a society. They generate, configure, and maintain social hierarchies in a society through *structural mechanisms* in terms of differential *socioeconomic positions* when the populations are stratified according to class, gender, race/ethnicity, income, education, occupation, and other characteristics. The structural determinants then operate through a set of *intermediary determinants* to produce differential health-compromising conditions. The intermediary determinants include factors of individual-level influences that produce health outcomes, such as material circumstances, psychosocial circumstances, behavioral and/or biological factors, and health system and community contextual factors. The CSDH framework highlighted the causal priority of the structural factors in generating differentials in health risks for differential health outcomes [8]. It stresses power as a critical factor shaping social hierarchies and thus conditioning health differences between groups. The CSDH framework can be used to explore the *structural* and *intermediary* factors of health inequities, including maternal health, and understand the underlying mechanisms or the causal chains between these factors.

The application of the CSDH framework might have an important role in addressing maternal health inequities in India, which requires an exploration and a clear understanding of the structural and intermediary factors and the mechanisms that link them to facilitate an effective and sustainable translation [9, 10]. Despite a large amount of published papers concerning maternal and reproductive health in India and some focusing on equity, it has been acknowledged that there is a lack of attempt to systematically map the documented sources of maternal health inequities in India [11]. The only study we found that mapped and summarized the social determinants of maternal health inequities in India [11] was limited to the analysis of five structural determinants. In this review, we aim to map and summarize the evidence on *structural* and *intermediary* factors of maternal health in India and understand their causal chains or mechanisms of influence by using the CSDH framework.

### Adapting the CSDH’s social determinants framework to maternal health

**Fig. 1:**
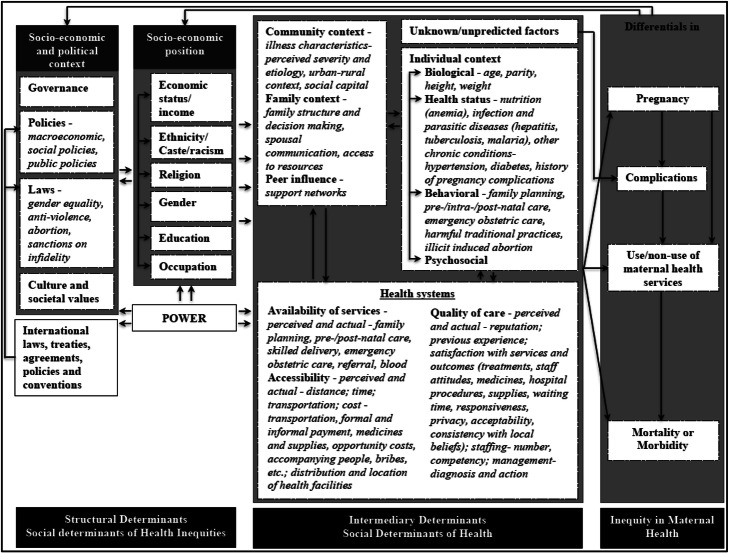
Conceptual framework of social determinants of maternal health

To achieve our study aim, we adapted the CSDH framework to maternal health (Fig. 1) by integrating two widely used frameworks on factors of maternal health, namely the 1994 Thaddeus and Maine’s “three-delay model” [12] (initially published in 1990) and the 1992 McCarthy and Maine’s framework on distant and immediate determinants of maternal death [13].

In this integrated framework (Fig. 1), we identify maternal health as a social phenomenon with outcomes that are influenced by contextual factors [11]. The contextual factors that create social hierarchies or stratifications in societies produce maternal health inequities [8, 11]. We further identify governance, policies and laws (national), cultural and social values, and international laws, policies, treaties, and conventions as the socioeconomic and political contexts that create socioeconomic hierarchies in societies in terms of social class, ethnicity/racism, gender, education, occupation, and income. This socioeconomic position operates through a set of intermediate factors that can broadly be categorized into individual-level factors (biological, health status, behavioral, and psychosocial), environmental factors (community, family, and peer influences), and health system factors to produce differentials in maternal health outcomes.

## Methods

We conducted a *scoping review* of published, peer-reviewed journal articles in two databases (PubMed and Science Direct), focusing on structural and intermediary factors in the framework that influenced maternal health outcomes in terms of “maternal mortality” and “maternal health service use” (Fig. 1). Scoping reviews are preliminary assessment of potential size and scope of available research literature conducted, often rapidly, to map the existing evidence base and synthesize knowledge pertaining to a topic, irrespective of study quality, and are useful when examining areas that are emerging, to clarify concepts and identify gaps [14–16]. We also searched for additional articles in a search engine, Google Scholar, and did a manual reference check of the identified review articles. We acknowledge that other aspects of maternal health outcomes, such as pregnancy and maternal morbidity, are also important. However, considering the study’s feasibility, mainly the manageability of data, we focused on these two outcomes and databases. Further, maternal mortality is used as a sensitive indicator of maternal health [17]. In a “Stage-III” country—such as India—of the “Obstetric transition” model (characterized by high MMR between 299-50 maternal deaths per 100,000 live births, variable fertility, and predominate direct causes of mortality with issues on access to and quality of care; see Souza et al*.* [18]), the access and utilization of the obstetric care service in particular is a crucial aspect of maternal health to achieve a significant reduction in maternal deaths. For this, we used the combinations of terms (maternal health, maternal health service, maternal health care utilization, reproductive health, antenatal care, postnatal care, institutional deliver*, home deliver*, skilled delivery, determinants, factors, equit*, equalit*, inequit*, inequalit*, and India) in the title, abstract, and keywords (Appendix 1).

### Inclusion and exclusion criteria

We included peer-reviewed journal articles of both quantitative and qualitative studies conducted after 2000 when policy attention turned to maternal health in India, particularly following the Millennium Development Goals in 2000 and a series of events after 2000 that shifted maternal mortality from a condition to a problem status [19]. Based on the *Population*, *Concept*, *and Context* criteria for inclusion of the Joanna Briggs Institute’s manual for scoping review [20], we included studies from India at the country, state, sub-state or population group level, and multi-country studies with India as one of the study countries which had data available for individual countries. We included studies that explored factors influencing the use of maternal health service or factors responsible for maternal deaths.

Regarding qualitative studies, we included ones that analyzed or explored factors influencing the use of maternal health service or factors responsible for maternal deaths covering any aspect of the conceptual framework (Fig. 1). Regarding quantitative studies, we included studies that analyzed at least three ANC visits, institutional or home deliveries, and postnatal care (PNC) use as outcome variables and used data for analysis from primary or secondary sources. We excluded review articles and quantitative studies analyzing fewer than three ANC visits, safe deliveries, skilled birth attendance due to reasons such as inconsistency in definitions of the terms (e.g., skilled birth attendants), or indicators (safe deliveries).

### Data extraction and analysis

We conducted a *narrative integrative synthesis* approach [21, 22] to summarize the structural and intermediary factors of maternal health in India and understand their mechanisms of influence. From the quantitative studies, we extracted information (based on the conceptual framework into data display matrices) on author(s), study year, study population(s), study variables, and results of associations (e.g., odds ratios at 95% confidence interval, *P* value < 0.05). This provided a meaningful summary of the study results [21]. For qualitative studies, we analyzed the articles thematically through an iterative process of reading and coding them based on the codes derived from the conceptual framework. For both types of studies, we added emerging factors not included in the framework. Data, from both types of studies, was summarized according to the main categories of the conceptual framework. This summarization involved synthesizing, analyzing, and interpreting data from the quantitative and qualitative studies to understand the mechanisms of influence of the factors identified.

## Results

### Type of studies included

**Fig. 2:**
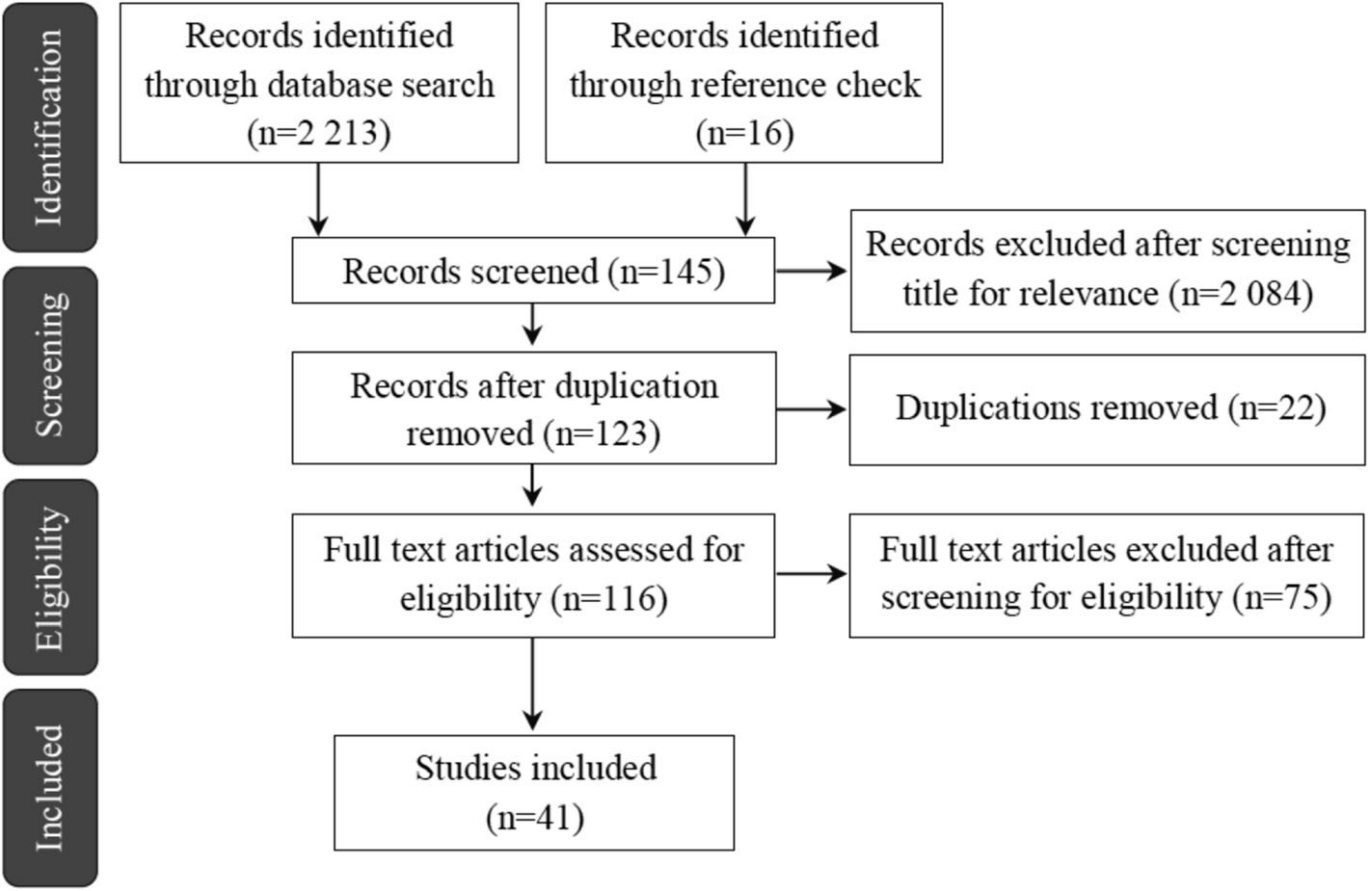
Study flow chart

From the original 2213 hits, we identified 25 full-text articles and an additional 16 articles from the reference check that met our study criteria; for details see Fig. 2. Eleven articles covered qualitative studies that focused on a district or sub-district region of the Indian states studied. For a summary of the quantitative articles included, see Table 1, and for details about the articles included, see Appendix 2.

**Table 1:** Characteristics of included quantitative studies

Outcome variable of analysis	***n***
Antenatal care	14
Institutional delivery	8
Postnatal care	2
Antenatal care and institutional delivery	3
Antenatal care and postnatal care	3
**Source of data**	
National Family Health Survey (NFHS)	14
NFHS Follow-up Survey	2
District-level Household and Facility Survey	7
Community-based Cross-sectional Study	4
Women’s Reproductive History Survey	1
Cohort Study	1
Key Informant Surveillance System	1
**Level of data for analysis**	
National^a^	16
Multistate^b^	3
State^c^	6
Other^d^	5

^a^Includes three multi-country, including national data for India; two each for India and state and India-urban and one each for India-urban and state-urban, India-urban-slum, India-urban-adolescent, India-rural, India-rural-adolescent, and India-adolescent

^b^Data analyzed is aggregated for more than one state

^c^Includes two for state-rural

^d^Iincludes two each for urban/city-slum and sub-district-rural and one district-rural

The quantitative studies most frequently focused on the influence of structural factors, such as economic status (including income), ethnicity/caste, religion, maternal education and occupation, husband’s education and women’s autonomy, and intermediary factors, such as maternal age, place of residence (urban-rural), parity, exposure to mass media, and history of complications. There were seven quantitative studies that studied the influence of health system variables [23–29], but they were limited to coverage, access, and infrastructure. Health systems-related factors were more extensively reported by the qualitative studies, most of which were on maternal deaths.

In the following sections, we aim to integrate the existing information from identified studies to provide an overview of social determinants of maternal health in India and their mechanisms of influence. First, we present the structural and intermediary factors of maternal mortality and maternal health service use in India based on the categories defined in the conceptual framework (Fig. 1). Then, we discuss their mechanisms of influence in the following section (the “Discussion” section).

### Social determinants of maternal health in India

We start with summaries of the findings under respective or sub-categories and then explain their relation to the use of maternal health service or maternal deaths.

### Structural factors

Poverty or economic status [23–25, 27–49], caste/ethnicity [23, 25, 29–42, 49–53], maternal education [23–25, 27–32, 37–40, 42–45, 47, 48, 54, 55], husband’s education [27, 29, 32, 44, 53], gender [31, 34–37, 47, 50, 54, 56], and religion [31, 32, 35, 43, 48, 56] were the most frequently associated structural factors of maternal health service use in India. Some studies also reported women’s employment status [31, 54] and husband’s occupation [55] as associated with or influencing maternal health service use.

#### Economic status

Poverty or economic status, often measured in terms of standard of living, household income, etc., has a significant impact on maternal health in India. Compared to the women from the poorest quintile, the odds ratios (OR) for the use of ANC and institutional delivery for the poorer, middle, richer, and richest groups in India were 1.3, 1.6, 2.2, and 3.7 [31]; and 1.6, 2.7, 3.6, and 11.4 [37], respectively. Similarly, studies in rural areas of Delhi and Karnataka confirmed the association of increasing household income with increasing use of maternal health service [53, 55].

#### Caste/ethnicity

Belonging to *socially backward castes* like *scheduled tribes* (ST), *scheduled castes* (SC), and *other backward castes* or *socially and economically backward castes* (OBC/SEBC) was associated with less use of maternal health service in India. Studies from Indian states reported a disproportionately high number of maternal deaths (50–80%) among the socially backward castes (in terms of population composition) [33, 34, 36, 41, 51]. Compared to women from castes other than SC/ST, women from SC and ST were less likely to have at least four antenatal check-ups (ORs 0.9 and 0.8, respectively) in 9 Indian states [29]. Similarly, the odds for not having institutional delivery among ST, SC, and SEBC women compared to other caste groups in Gujarat were 2.6, 1.3, and 1.3, respectively [39].

#### Education

Women’s education was consistently found to be associated with the use of maternal health service in India. The ORs for the use of ANC and PNC among women who had primary, secondary, and high school-level education compared to illiterate women were 1.5, 1.9, and 2.7; and 1.3, 1.7, and 2.4, respectively [31]. Studies also found that if the husband was educated, this was likely to increase his spouse’s use of maternal health service in India [27, 29, 32, 44, 53].

#### Gender

Gender, particularly the low status of women (in terms of autonomy and decision-making) and son preference, influenced maternal health in India [31, 34–37, 47]. Women with greater autonomy were more likely to use maternal health service in India [31, 37, 47, 56]. However, studies conducted among adolescents did not find any influence of autonomy on maternal health use [27, 28]. Women in West Bengal who participated in household decision-making were four times more likely to have institutional deliveries than those not participating in household decision-making [56]. Gender norms that accept violence against women also influenced maternal health status in India [54, 56]. Women experiencing any form of violence during pregnancy were less likely to have institutional deliveries in Maharashtra (OR–0.6) and Uttar Pradesh (OR–0.7) compared to women not experiencing any violence [56].

#### Religion

Compared to Hindu women, Muslim women were less likely to have more than four ANC visits in 9 Indian states (OR–0.9) [29] and more likely to deliver at home in rural Delhi (OR–6.2) [53]. Christian and Sikh women, on the other hand, were 5.5 and 18.5 times, respectively, more likely to have more than three ANC visits in urban slums of eight Indian cities compared to Hindu women [43].

#### Culture

Even though culture was not explicitly identified or analyzed as a factor influencing maternal health in India, several cultural practices were found to be influential. For example, the culture of *Purdah* widely practised by Muslims in south India, which restricts women’s freedom of movement outside the household, limited their use of maternal health service from male service providers [57]. Similarly, the culture of early marriage in India was observed as having a crucial influence on maternal health as the majority of maternal deaths occurred among young women in India [24, 41, 50]. Studies found that women married at an early age in India were less likely to use ANC and institutional deliveries [32, 39, 58].

Other cultural practices like women going to their mothers’ home during pregnancy, especially for the first pregnancy in Tamil Nadu, had a positive influence on maternal healthcare [46]. But, in some cases it led to a delay in seeking care, particularly if there was a cultural norm for the mothers to seek decisions about their married daughter’s health from her in-laws and husband [50].

### Intermediary factors

#### Community context

Community-related factors that were found to influence maternal health service use in India were place of residence [29, 30, 32, 37, 39, 40, 42]; higher concentration of wealth and education at the community level [29–31] (particularly for women [29, 30]) and of large family [31] and caste, particularly SC/ST [30]; factors influencing accessibility of health service, namely, distance to health facilities [25, 34, 36], village connected by all-weather road [23], and availability of transportation [41, 52]; and migration status or duration of residency [24].

Rural residence was consistently associated with a low use of maternal health service in India, while urban residence was associated with a high use. Rural women in India were less likely to have ANC visits checkups (OR–0.8), institutional deliveries (OR–0.5) [37], and postnatal checkups (OR–0.8) [31] compared with their urban counterparts. Compared to communities with 0–25% illiterate women, those with more than 50% and 26–50% illiterate women were less likely to have more than four ANC visits in nine Indian states (ORs 0.8 and 0.9, respectively) [29]. Similarly, the communities with more than 50% SC/ST were less likely to have a full ANC (OR–0.9) compared with communities with 0–25% SC/ST in eight Indian states [30]. Compared with women who resided less than 2 km away from health facilities, the odds of delivering at institutions was 0.38 for women residing more than 2 km away in urban slums of Gujarat [23]. Availability of an all-weather road increased the likelihood of institutional delivery (OR–1.2) in rural Uttar Pradesh [25]. Lack of transportation was also reported to be a major barrier for maternal health service use [41, 52]. Migration status or duration of residency in terms of annual migration rate > 25% was also found to be associated with high home delivery (OR–1.6) in Mumbai, particularly in slums [24].

#### Family or household context

Family structure or size, particularly joint/extended and large family size, was found to be associated with a higher use of maternal health service [55, 59]. Compared with women residing in nuclear and small families, the likelihood for PNC use was higher for those residing in joint (OR–1.5) and large families (OR–2) [55]. In nuclear families, women with better relationships with their husbands were more likely to have ANC check-ups and institutional deliveries [60].

#### Individual context

At the individual level, factors that were associated with or influenced maternal health service use in India were categorized as biological or demographic factors, health status- or need-related factors, awareness-related factors, behavioral or service use-related factors, and psychosocial factors.

##### Biological or demographic factors

Women’s age at marriage [32, 39, 58], maternal age at childbirth [24–26, 29, 30, 33, 34, 37, 39, 40, 42, 50, 51], and parity [23–25, 27–31, 37, 38, 40, 43, 45, 48] were found to influence maternal health service use in India. Young age at childbirth and high birth order or parity were consistently found to be associated with a lower use of maternal health service in India. Studies also found that women married at an early age were found to use ANC and institutional deliveries less in Rajasthan and Gujarat [32, 39]. Compared with women who were married before 18 years of age, those married between 18 and 21 years and after 21 years of age were 2.4 and 4.5 times more likely, respectively, to have a full ANC in Rajasthan [32]. Compared with women aged 15–24 years, the odds of delivering in an institution were 1.19 and 2.44 for women aged 25 to 35 years and more than 35 years, respectively [37]. Women with two or more children were less likely to use ANC (OR–0.8) and have an institutional delivery (OR–0.7) than women with one child in India [37].

##### Health status- or need-related factors

History of complications [25] and adverse pregnancy outcomes [55] were the health need-related factors found to influence maternal health service use in India. A study in rural Uttar Pradesh found that women who had experienced complications in previous pregnancies/deliveries were three times more likely to deliver in health institutions than those who had no such history [25]. A study in rural Karnataka also found that women with a history of neonatal deaths were 7.5 times more likely to use PNC service than those without such a history [55].

##### Awareness-related factors

Exposure to mass media [31, 40, 42, 44, 47] and maternal health messages [29, 30, 48, 55, 56] were found to influence maternal health service use in India. Compared with women without any exposure to mass media, women who had been exposed were more likely to use ANC (OR–1.4) and PNC (OR–1.3) in India [31]. Studies found that women who had not heard or seen any messages about ANC were less likely to use ANC service (OR–0.7) than women who had heard or seen messages on ANC in nine Indian states [29]. Similarly, women who had a high level of awareness of PNC service were 2.4 times more likely to use them than those who had a low level of awareness in rural Karnataka [55].

##### Service use-related factors

The use of ANC was found to influence institutional delivery and PNC [23–25, 29, 38, 44, 45, 48, 55], while institutional delivery or delivery by skilled birth attendants influenced PNC use [27, 29, 45, 48, 55] in India. Women using ANC were more likely to having institutional deliveries [23–25, 38, 44] and PNC [29, 45, 48, 55] in India. Likewise, women delivering in an institution were more likely to undergo a PNC check-up [29, 45, 48, 55].

##### Psychosocial factors

Psychosocial factors influencing maternal health service use in India included perception of care, i.e., benefits of maternal care [34, 36, 52] and quality [36, 46, 52, 61], fear of health interventions like surgery or caesarean sections [36, 46, 52, 61], and wanted pregnancy [38, 47]. About 34% of women in India reported not delivering in health institutions because they did not think it necessary, while an additional 6.5% did not due to a lack of knowledge [5]. Societies’ perception of considering pregnancy and childbirth as normal phenomena rather than a life-threatening situation was the reason why women did not seek healthcare in some cases in rural Rajasthan [34]. Communities’ perceived attitudes towards supernatural healing also reduced their readiness to seek care in a health facility in Odisha [52]. Women did not consider ANC beneficial as they observed other women in the communities delivering normally even without ANC [52]. Women’s experience of previous normal home deliveries without complications also renders them likely to perceive pregnancy and childbirth as normal phenomena [36]. Studies reported instances where women considered home delivery safer as they believed that caesarean sections were performed unnecessarily in hospitals and that they lead to a greater hazardous risk [52].

Fear of caesarean sections at hospitals also led women to opt for home delivery and avoid surgery in urban Mumbai and rural Kerala, respectively [36, 61]. Likewise, fear of the hospital environment and unfamiliar surroundings and fear of being alone during childbirth influenced women not to opt for institutional delivery in Odisha and to choose the private sector in Kerala [36, 52]. In contrast, permitting a birth companion was seen as one of the reasons why women preferred Primary Healthcare Centers (PHCs) for deliveries, especially for first deliveries, in Tamil Nadu [46].

Studies reported that families’ negative perceptions of care (quality) provided in government hospitals made them reluctant to take women there despite their knowledge about the free service provided in the hospitals [50]. Similarly, a perception of better care at private hospitals and PHC compared with public hospitals and referral government hospitals was the reason why families chose private facilities and PHC in Mumbai and Tamil Nadu, respectively [46, 61].

Compared with wanted pregnancies, the odds were 2.3 for not having adequate ANC for unwanted pregnancies in rural areas of four Indian states [47]. Women with wanted pregnancies were also more likely to have institutional deliveries (OR–1.9) compared with those with unwanted pregnancies in rural Andhra Pradesh [38].

#### Health system

Studies reported issues related to availability, accessibility, and/or quality of maternal health service, often resulting in maternal deaths [33–36, 41, 46, 50, 52, 62, 63]. For instance, about 15–60% of maternal deaths in five Indian states were due to delays in receiving appropriate and adequate care in health facilities [33, 50].

Studies reported that health facilities in India, even the Community Health Centers that are regarded as First Referral Units, often lacked requisite expertise and facilities in terms of competent staff or specialists, equipment, and supplies [33–35, 41, 50, 62, 63]. Studies frequently highlighted a critical lack of blood in India that led to delayed or inadequate blood transfusions in emergencies [36, 41, 63]. The lack of services often resulted in a high number of referrals of women with an obstetric emergency that in turn led to further delay in receiving appropriate care [33, 35, 41, 46, 63].

Studies documented a high number of referrals in search of maternal care in India that were responsible for maternal deaths [33–36, 41, 62, 63]. The referrals were often unassisted, and the responsibility for arranging vehicles also rested with the families who then spent a significant amount of time in arranging a vehicle and money for the travel and the treatment costs at health facilities, which were often at great distances [34, 41]. Instances of maternal deaths were also reported in rural areas of Madhya Pradesh [41], Rajasthan [34], and Uttar Pradesh [35] when women were unable to reach a referred health facility.

Inappropriate treatment and the negative attitude of health professionals towards women and families were reported as responsible for families hesitating to seek healthcare and receive appropriate maternal healthcare in India [35, 41, 50]. Studies also reported instances of discrimination based on caste or other characteristics, such as dirty or ragged clothing [35, 50, 52], and verbal and physical abuse of women by public health facilities staff in Madhya Pradesh and Uttar Pradesh [35, 41]. On the other hand, the attentive, responsive, courteous, empathetic, and respectful attitude of PHC staff, from the doctors to the cleaning staff, emerged as the key factor for women to choose PHC even over referral hospitals and private hospitals for birthing care in Tamil Nadu [46]. The study also highlighted that this mattered more to women than the technical competence of the providers.

Health professionals were also reported as not adhering to standard protocols while providing obstetric care or referrals to all levels of facilities across Indian states [41, 62]. Studies also highlighted the cases of referrals when the women were not even stabilized or given any first aid before referrals [36, 41]. The auxiliary nurse-midwives (ANM) based at PHCs and health sub-centers in Madhya Pradesh were reported as not visiting villages to provide ANC [41]. The study also reported instances of improper demands for payment and corruption at all levels of the health system in Madhya Pradesh [41].

Two studies reported that ANC was found to be either absent or inadequate [34, 41]. Postpartum care was also found to be completely absent both in the facility and in the community in rural Rajasthan [34]. The study also reported that despite anemia being responsible for about 20% of maternal deaths in India, it remained undetected during ANC examinations [34].

Table 2 provides an overview of the structural and intermediary factors of maternal mortality and maternal health service use in India as identified in this review.

**Table 2:** Overview of structural and intermediary factors of maternal health in India

Structural factors	Intermediary factors		
		Health systems	
▪ Economic status ▪ Caste/ethnicity ▪ Gender - Women’s autonomy - Son preference - Exposure to violence ▪ Religion ▪ Culture - Early marriage ▪ Maternal education ▪ Women’s employment ▪ Husband’s education ▪ Husband’s occupation ▪ Power ▪ Policy gaps - Health worker not seeing pregnant women in parental homes - Citizenship issues for migrants - Two-child norm and policies promoting sterilizations	**Community context** ▪ Place of residence ▪ Distance ▪ Transportation **Family/household context** ▪ Family type and size ▪ Quality of relationships **Individual** ▪ ***Biological or demographic*** - Age at marriage - Age at childbirth - parity ▪ ***Health need*** - Anemia - History of complications (previous pregnancies) - History of adverse pregnancy outcomes (previous pregnancies) ▪ ***Knowledge*/awareness** - Awareness of danger signs and maternity entitlements - Exposure to mass media and maternal health messages ▪ ***Behavioral*** - Use of antenatal care, institutional delivery ▪ ***Psychosocial*** - Fear of caesarean section and surgery - Perception of benefits - Perception of care - Desired or wanted pregnancy - Permitting birth companion	▪ ***Availability of services*/*facilities*** - Infrastructure, equipment, blood, and health specialist - Lacking or no proper antenatal care - Care for anemia - Postnatal care at communities and health facilities - Abortion services at public facilities - Lacking or poor provider’s skill and competence: doctor, nurse, auxiliary nurse-midwife (ANM), Accredited Social Health Activist (ASHA) - Lack of fuel for ambulance ▪ ***Quality of care*** *Staff attitude and behavior* - Poor adherence to standards and protocols - Negligence or lack of care by health worker, including ASHA/ANM not visiting villages - Non-responsive and disrespectful behavior - Discrimination by health workers based on social status - Physical and verbal abuse by health workers - Lack of priority for maternal healthcare - Tendency of transfer of blame in hierarchy - Low motivation *Referral* - Unnecessary or irrational referral - Multiple referrals - No appropriate care before referral - Unassisted referrals	*Other* - Longer waiting time at government hospitals - Illegal demand for money: ambulance - Lack of proper care in private hospitals ▪ ***Accessibility*** - Cost associated with healthcare - Lack of ambulance or transport after referral - Cost associated with referral - Organization of services (Basic & Comprehensive Emergency Obstetric Care): challenge for referral - Illegal demand for money ▪ ***Administrative*,*managerial*,*governance*** - Ambulance not authorized to drive to another village/hospital - Organization and supervision of ambulance services - Lengthy administrative procedures - Lack of proper monitoring and supervision, including no proper maternal death reviews - Lack of accountability mechanisms - Non-issuance of Below Poverty Line cards - Documentary proof of poverty ▪ ***Working environment*** - Understaffed and over-pressured staff - Lack of supportive infrastructure for health workers, e.g., road/transport

## Discussion

A number of structural and intermediary factors were identified as influencing maternal health service use and maternal mortality in India. In line with Sanneving et al., economic status, caste/ethnicity, education, gender, and religion emerged as the most pertinent structural factors of socio-economic position. We also identified culture (the characteristics or “way of life” of a particular group involving language, social habits, religion) as a structural factor of socio-economic and political context. Similarly, place of residence, maternal age at childbirth, parity, women’s exposure to mass media, and maternal health messages were the most pertinent intermediary factors. Women, particularly from low economic status, socially backward and marginalized castes like SC/ST, uneducated, Muslim, rural, childbearing at a young age, and with two or more children, who had hardly any exposure to mass media or maternal health messages, seemed to be more disadvantaged in terms of maternal health service use or more prone to maternal deaths in India.

Our findings are consistent with other reviews [64–66] and studies [67–70] conducted outside the Indian context for—economic status/household wealth/household income [64–70], ethnicity [68–70], maternal education [64, 66–70], women’s autonomy (gender) [66, 67], religion [69, 70], place of residence [64, 66, 68–70], maternal age at childbirth [68], parity [64, 66, 67, 69], and women’s exposure to mass media [66, 67, 69].

### Mechanisms of influence

We observed that the structural factors influenced intermediary factors for maternal health service use or maternal deaths, either independently or in association with other structural factors. This influence occurred at multiple contexts, e.g., community, household, and individual contexts. We also observed the health system as a crucial intermediary factor which not only influenced the maternal health outcomes directly but also influenced other intermediary factors at the individual level, such as psychosocial and behavioral factors. Lack of accountability was identified as one of the major factors negatively influencing the health system.

In the following sections, we discuss the influence of structural factors to produce maternal health outcomes. Then, we discuss the health system as an important intermediary factor and its accountability-related issues. As the CSDH framework underlines, we also observed power as a crucial structural factor of maternal health. We primarily use the studies that we reviewed to explain the influence and the relations between the factors specific to the Indian context.

#### Influence of structural factors

Culture played out at socio-economic/political context to influence maternal health service use or maternal deaths in India, often related to religion, caste/ethnicity, and gender norms. The socio-economic position of women, and in particular their economic status, caste/ethnicity, religion, and education, appears to have an effect on maternal health service use or maternal deaths. Saxena et al. found that being poor was independently associated with less use of maternal health service, regardless of caste or place of residence [39]. Caste was also found to have a strong independent effect on maternal health service use by the same study [39]. SC/ST followed by OBC/SEBC is among the most socio-economically disadvantaged groups in India and distinguished by economic poverty [11, 39, 50]. Religion overlaps with the caste system in India, with more than 40% of the Muslim community belonging to the OBC group [11]. Muslims, in general, are socioeconomically disadvantaged in India, with a higher proportion of the Muslim population living under the poverty line (35.4%) compared with the Hindu population (29.7%) and other religions (19.4%) in 2009–2010 [71]. Maternal education particularly benefits women by providing greater access to information about the risks of pregnancy and childbirth, existing healthcare services and entitlements, and making decisions about healthcare [31, 57].

We observed that structural factors influence each other to have an effect on intermediary factors. Solar and Irwin mentioned that economic status influences health indirectly by mediating more proximal factors in the causal chain of health production, e.g., health behaviors through education [8]. Education also enables women to exercise greater autonomy inside and outside the household to travel and seek care, and communicate about their health problems, demand services, and make decisions to use modern healthcare [57].

Structural factors exert an influence at multiple levels. For example, at the individual or household levels, economic status was directly linked to out-of-pocket expenditure to access maternal healthcare. Studies reported families often taking a long time to arrange money to meet such expenses, and in extreme situations they avoided seeking treatment [34, 35, 72]. The advantage of the higher economic status of joint and large families accounting for higher maternal health service use in India [59] also shows the influence of economic status at the family or household level. The associations of maternal health service use with the community’s concentration of wealth, women’s education, and caste demonstrate the influence of such structural factors at the community level in India.

#### Health system—an important intermediary factor

Solar and Irwin emphasized that the health system is an independent intermediary factor in itself as it can influence differential exposure and vulnerability to health inequities as well as directly address them [8]. We also observed that the health system had a direct influence on maternal health service use among women in India.

Health system-related factors particularly influenced the women’s psychosocial factors. Studies found that women usually acquired information related to cost and quality of care from their past experiences or from relatives, friends, and neighbors [34, 46, 61]. One of the major reasons why women did not seek appropriate maternal healthcare in India was their lack of awareness about care during pregnancy, obstetric risks, and maternal entitlements [34, 46, 50, 72]. One of the main reasons why women lacked such information in India was the lack of ANC [72].

Health system-related factors also influenced women’s access to maternal health service in India directly or independently. For example, as facilities did not maintain critical emergency supplies of blood, the responsibility of arranging blood lays with the families, which often involved large sums of money [63, 72]. In contrast, the health system through policy interventions like *Janani Suraksha Yojana* (JSY)^1^ also enhanced women’s access to maternal health service through conditional cash incentives [73–76]. However, policy norms requiring documentary proof of poverty that are not issued regularly to the right beneficiaries, and conflicting policies that do not consider entitlements for vulnerable groups such as migrants, women, and girls with more than two children, etc. were reported as hampering women from accessing services [72].

Health system performance is influenced by various factors that are both external and internal to the system. Examples of external factors include an unsupportive environment in terms of lack of road/transport for Accredited Social Health Activists (ASHAs^2^)/ANM to make community visits [41]. Studies predominantly reported multiple issues within the health system that constrained the provision of maternal health service in India. For example, the health facilities lacked competent staff to identify and manage obstetric complications due to vacancies, staff absenteeism, or a lack of adequate training and supervision [27, 33]. The inappropriate treatment of women in emergencies by health professionals was sometimes due to understaffing or staff under too much pressure to pay enough attention to a woman or their engagement in administrative tasks [62, 72]. Studies reported such problems of health systems were due to lack of priority for maternal healthcare over other programs such as polio, sterilization, etc.; lack of proper monitoring and supervision; and lack of accountability [41, 62, 72, 78].

Studies critically highlighted the lack of accountability of the health system in India as a reason for failure to ensure proper essential obstetric care [41, 62, 78]. Accountability issues were more distinct in terms of irrational and unassisted referrals, irresponsible and disrespectful behavior, and the negative attitude of health workers, especially towards poor and marginalized women, and corruption and illegal demands for money [41, 62, 78]. The health system also lacked accountability mechanisms like a complaint procedure or mechanism for redress, administrative control, performance assessment, and disciplinary procedures [41, 62, 72, 78].

#### Power—an important structural factor of maternal health inequities

The CSDH framework highlighted the central role of *power*, in terms of the domination of certain groups over the others, in generating health inequities by creating social hierarchies in societies [8]. We also observed similar power issues in terms of domination over certain disadvantaged groups such as women, SC/ST, and Muslims in Indian society.

SC/ST is among the most socially marginalized and isolated groups from the rest of society in India [11, 39, 50]. Muslims also have lower literacy rates than the national average and under-representation in governmental and political positions [11]. Women, especially from such socially backward classes, probably do not have the power to enforce their rights to have access to and control of economic, political, and cultural institutions, leading to their exclusion from or incapacity to influence public policies or decisions affecting their interests [8, 79]. As Berlan and Shiffman mentioned, such power asymmetries arising out of social contexts influence health providers’ behavior [80]; undesirable behaviors of health workers against socially disadvantaged groups were reported in Odisha [34, 35, 52] and Uttar Pradesh [78] in terms of physical abuse or violence, caste-based discrimination, and disrespect.

We observed issues of power in terms of asymmetrical power relations in the hierarchical health system of India. Studies reported that blame is often laid on to staff at lower levels of the health system in India for misdeeds or inappropriate healthcare [41, 62]. Health workers often control information that has implications for the healthcare monitoring and accountability systems [78]. Owing to their technical knowledge about health, health providers usually control the healthcare decisions that have implications for people’s access to and experience of healthcare [81, 82]. Several studied cases revealed that health professionals often had the power to make decisions based on their own judgment and interest rather than following a standard protocol, e.g., in cases of multiple irrational referrals [36, 41, 62, 72, 78].

Solar and Irwin emphasized that to address health inequities, it would require understanding how power operates in multiple dimensions of economic, social, and political relationships to clarify the causal processes that underlie them. The studies we identified in our study, however, were not explicitly addressing power. Hence, we urge for more studies to explore and understand the issues of power responsible for differential access to maternal healthcare for different socioeconomic groups in India.

## Limitations of the study

A major limitation of our study pertains to its scope. Given the very broad scope of the integrated framework and considering the feasibility of the review in terms of the manageability of the data, we focused on factors of maternal health service use and maternal deaths. In this *scoping* review, we were therefore not able to include other relevant aspects of the framework (such as governance, international laws, policies, and treaties) and maternal health (e.g., unsafe abortions, anemia, and maternal morbidity). This could have also been due to the lack of studies on these topics or the limitations of our search strategy (scoping review and inclusion and exclusion criteria). However, we have been able to indicate the areas for further research pertaining to the framework and maternal health.

There were not enough studies for all sets of indicators and sub-population groups. For example, even though the majority of maternal deaths took place in the postnatal period, we found very few quantitative studies analyzing the association of PNC with the potential factors. We recommend more studies to explore the variables influencing the use of PNC service in Indian contexts and analyze their associations. Comparisons could not be done explicitly for any given indicators for any states and between rural and urban areas of the states mainly because of three reasons: lack of uniform use of the same indicators, focus on different settings of the study areas, and not all outcome variables for all states or population groups are available.

Our analysis of the relationships or mechanisms of influence among the different variables in our study has been restricted to the evidence available in the limited included literature. Moreover, not all of the relationships found among the variables in our study are empirically established. Nevertheless, we have tried to establish a more comprehensive view of the linkages between the variables by synthesizing evidences from the quantitative and qualitative studies. We have been able to identify a wide range of factors in India as the quantitative and qualitative studies complemented each other, which were otherwise not covered by either type of study. For example, health service delivery system-related factors emerged more explicitly from the qualitative studies, while some factors like maternal education, exposure to mass media, family structure, etc. were identified from quantitative studies. By combining these different types of studies, we were able to identify and discuss some crucial aspects of maternal health inequities, such as issues of power and accountability.

## Conclusion

With the aid of the framework, we were able to integrate the existing information from quantitative and qualitative studies to produce a more comprehensive picture of factors of maternal mortality and maternal health service use in India in terms of structural and intermediary factors and their mechanisms of influence. This review sets an example of the potential that the framework holds to provide comprehensive analyses of determinants of maternal health service use and maternal deaths, and the interlinkages between them for any given context, even beyond the Indian context. The analyses can also be focused on any one or selected aspects of the framework (e.g., health system, structural determinants, intermediary determinants, etc.) or on any specific communities or population group. Such analyses would be helpful to provide more focused recommendations for context-specific policy and program interventions. While our review represents a more comprehensive view of the factors of maternal health in India, we acknowledge that there are state- and community-specific variations of the factors and other factors that we could not address. So, we urge more state-specific or even community-specific research to be done with the analysis focused on context-specific policy implications to address maternal health inequities in such populations as well as to review aspects such as socio-economic contexts and caesarean sections. In particular, more qualitative research is needed to explore all possible structural and intermediary factors of influence and the mechanisms of their influence.

## Data Availability

The datasets used and/or analyzed during the current study are available from the corresponding author on reasonable request.

## Appendixes

**Table 3:** Search strategy

Search#	Search terms	Where (keywords)	Total hits	Date searched
**PubMed**				
1	(((((((((((maternal health[Title/Abstract]) OR maternal health care utilization[Title/Abstract]) OR maternal health services[Title/Abstract]) OR reproductive health[Title/Abstract]) OR antenatal care[Title/Abstract]) OR postnatal care[Title/Abstract]) OR skilled delivery[Title/Abstract]) OR skilled attendance[Title/Abstract]) AND determinant*[Title/Abstract]) OR equit*[Title/Abstract]) OR equalit*[Title/Abstract]) AND India[Title/Abstract]	title/abstract	442	02–04 April 2016
**ScienceDirect**				
2	"maternal health" OR "maternal health care utilization" OR "maternal health services" OR "reproductive health" OR "antenatal care" OR "postnatal care" OR "skilled delivery" OR "skilled attendance" AND determinant* OR equit* OR equalit* AND India.	n.a.	1,712	06–08 April 2016
**Google Scholar**				
3	'maternal health' 'determinants' 'India'	title	7	06 April 2016
4	'maternal health' 'factors' 'India'	title	7	06 April 2016
5	'skilled delivery' 'determinants' OR 'factors' 'India'	title	4	06 April 2016
6	'antenatal care' 'determinants' OR 'factors' 'India'	title	11	06 April 2016
7	'postnatal care' 'determinants' OR 'factors' 'India'	title	4	06 April 2016
8	'institutional delivery' 'determinants' OR 'factors' 'India'	title	1	06 April 2016
9	'institutional deliveries' 'determinants' OR 'factors' 'India'	title	1	06 April 2016
10	'home deliveries' 'determinants' OR 'factors' 'India'	title	0	06 April 2016
11	'home delivery' 'determinants' OR 'factors' 'India'	title	0	06 April 2016
12	'maternal health care utilization' OR 'maternal health services' 'determinants' OR 'factors' 'India'	title	4	06 April 2016
13	'reproductive health' 'determinants' OR 'factors' 'India'	title	15	06 April 2016
14	'maternal health' AND 'inequalities' AND 'India'	title	5	06 April 2016
15	'maternal health' AND 'inequities' AND 'India'	title	0	06 April 2016
16	Reference check	title/abstract	n.a.	08–11 April 2016

**Table 4:** Studies included

Author(s)	Title	Study year	Indicator(s) included	Study area(s)	Source(s)
Alcock et al. [61]	Examining inequalities in uptake of maternal health care and choice of provider in underserved urban areas of Mumbai, India: a mixed methods study	2011–2013	Qualitative	Maharashtra—Mumbai two informal settings	PubMed
Allendorf [60]	The quality of family relationships and use of maternal health-care services in India	2010	Institutional delivery	Madhya Pradesh	PubMed
Allendorf [59]	Going nuclear? Family structure and young women’s Health in India, 1992-2006	2012	ANC^1^	India	Reference check
Bhanderi and Srinivasan [23]	Utilization of maternal health services and determinants of skilled care during delivery in slums of Gujarat, India	2015	Institutional delivery^2^	Gujarat—urban slum in Rajkot city	Google Scholar
Chattopadhyay [56]	Men in maternal care: evidence from India	2012	Institutional delivery	India, Uttar Pradesh, West Bengal, and Maharashtra	PubMed
Chauhan [47]	Antenatal care among currently married women in Rajasthan, India	2012	Full ANC^3^	Rajasthan	ScienceDirect
Das et al. [24]	Prospective study of determinants and costs of home births in Mumbai slums	2010	Home delivery	Mumbai—slums, six municipal wards	Reference check
Dikid et al. [48]	Maternal and perinatal death inquiry and response project implementation review in India	2009	Qualitative	Bihar, Rajasthan, and Orissa	PubMed
George A [62]	Persistence of high maternal mortality in Koppal District, Karnataka, India: observed service delivery constraints	2004	Qualitative	Karnataka—Koppal district	ScienceDirect
Godha et al. [58]	Association between child marriage and reproductive health outcomes and service utilization: a multi-country study from South Asia	2013	≥ 4 ANC, institutional delivery	India (and Bangladesh, Nepal, and Pakistan)	Reference check
Goli et al. [40]	Pathways of economic inequalities in maternal and child health in Urban India: a decomposition analysis	2013	< 3 ANC, not an institutional delivery	India—urban	Google Scholar
Hazarika [39]	Women’s reproductive health in slum populations in India: evidence from NFHS-3	2009	≥ 3 ANC	India—eight cities, urban slums	Reference check
Iyengar et al. [30]	Pregnancy related deaths in rural Rajasthan, India: exploring causes, context, and care-seeking through verbal autopsy	2002–2003	Qualitative	Rajasthan—a block	Reference check
Jat et al. [41]	Factors affecting the use of maternal health services in Madhya Pradesh state of India: a multilevel analysis	2011	PNC^4^	Madhya Pradesh	Google Scholar
Jat et al. [50]	Socio-cultural and service delivery dimensions of maternal mortality in rural central India: a qualitative exploration using a human rights lens	2011	Qualitative	Madhya Pradesh—Khargone district	Reference check
Jayanthi et al. [42]	Primary health centres: preferred options for birthing care in Tamil Nadu, India, from users’ perspectives	2012	Qualitative	Tamil Nadu	Reference check
Jeffery P and Jeffery R [31]	Only when the boat has started sinking: A maternal death in rural north India	2002–2005	Qualitative	Uttar Pradesh—Muslim village in rural Bijnor district	ScienceDirect
Jihesh and Sundari Ravindran [32]	Social and health system factors contributing to maternal deaths in a less developed district of Kerala, India	2010–2011	Qualitative	Kerala—Wayanad district	Google Scholar
Koski et al. [54]	Physical violence by partner during pregnancy and use of prenatal care in rural India	2011	At least 3 ANC	India—rural	Reference check
Kumar and Mohanty 2011	Intra-urban differentials in the utilization of reproductive healthcare in India, 1992-2006	2011	ANC^3^	India—urban	Reference check
Mahapatro [52]	Equity in utilization of health care services: perspective of pregnant women in southern Odisha, India	2011–2012	Qualitative	Odisha—Gajam	PubMed
Mahapatro [33]	Utilization of maternal and child health care services in India: does women's autonomy matter?	2012	Full ANC^3^	India	Reference check
Nair et al. [34]	What influences the decision to undergo institutional delivery by skilled birth attendants? A cohort study in rural Andhra Pradesh, India	2012	Institutional delivery^5^	Andhra Pradesh—rural	PubMed
Pathak et al. [6]	Economic inequalities in maternal health care: prenatal care and skilled birth attendance in India, 1992-2006	1992–2006	ANC^6^	India, Uttar Pradesh, Maharashtra, Tamil Nadu	Google Scholar
Paudel et al. [55]	Determinants of postnatal maternity care service utilization in rural Belgaum of Karnataka, India: A community based cross-sectional study	2014	PNC services	Karnataka—rural Belgaum Taluka	Google Scholar
Prakash R & Kumar A, [49]	Urban poverty and utilization of maternal and child health care services in India	2013	ANC^7^	India—urban, eight EAG^11^, Maharashtra, and Tamil Nadu	Reference check
Ram and Singh [25]	Is antenatal care effective in improving maternal health in rural Uttar Pradesh? Evidence from a District Level Household Survey	2006	Institutional delivery	Uttar Pradesh—rural	Reference check
Roy et al. [26]	Determinants of utilization of antenatal care services in Rural Lucknow, India	2013	3 ANC	Uttar Pradesh—Lucknow district	Google Scholar
Sahoo et al. [53]	Do socio-demographic factors still predict the choice of place of delivery: a cross-sectional study in rural North India	2015	Home delivery	Delhi—two villages	ScienceDirect
Saxena et al. [35]	Inequity in maternal health care service utilization in Gujarat: analyses of district-level health survey data	2013	≥ 3 ANC, institutional delivery	Gujarat	PubMed
Singh et al. [27]	Assessing the utilization of maternal and child health care among married adolescent women: evidence from India	2012	Full ANC^3^	India—adolescent	PubMed
Singh et al. [28]	Determinants of maternity care services utilization among married adolescents in rural India	2012	Full ANC^3^	India—rural, adolescent	PubMed
Singh et al. [43]	The consequences of unintended pregnancy for maternal and child health in rural India: evidence from prospective data	2013	Use of ANC^6^	Bihar, Jharkhand, Maharashtra, Tamil Nadu	Reference check
Singh et al. [29]	Factors associated with maternal healthcare services utilization in nine high focus states in India: a multilevel analysis based on 14 385 communities in 292 districts	2013	≥ 4 ANC, PNC^8^	9 states	PubMed
Singh et al. [44]	Utilization of maternal health care among adolescent mothers in urban India: evidence from DLHS-3	2014	Full ANC^3^, PNC^9^	India—urban, adolescent	Reference check
Singh et al. [45]	Factors influencing antenatal care services utilization in Empowered Action Group (EAG) States, India: a spatial and multilevel analysis	2015	Full ANC^3^	India—eight EAG states	Google Scholar
Subha Sri et al. [27]	An investigation of maternal deaths following public protests in a tribal district of Madhya Pradesh, central India	2010	Qualitative	Madhya Pradesh—Barwani district	ScienceDirect
Tey and Lai [36]	Correlates of barriers to the utilization of health services for delivery in South Asia and Sub-Saharan Africa	2013	Institutional delivery^10^	India—and Bangladesh, Pakistan, Kenya, Nigeria, and Tanzania	Reference check
Viegas Andrade et al. [38]	Antenatal care use in Brazil and India: scale, outreach and socioeconomic inequality	2012	> 4 ANC	India	PubMed, ScienceDirect
Vijayshree et al. [63]	“She was referred from one hospital to another”: evidence on emergency obstetric care in Karnataka, India	2011	Qualitative	Karnataka	Reference check
Yadav and Kesarwani [46]	Effect of individual and community factors on maternal health care service use in India: a multilevel approach	2015	Full ANC^3^, PNC^4^	India	Google Scholar

^1^> 3 ANC visits, beginning in the first trimester of pregnancy

^2^Referred to as skilled delivery care in this study

^3^At least 3 ANC, at least 2 TT during pregnancy or 1 TT during pregnancy, and 1 during 3 years prior to the pregnancy received iron and folic acid tablets for at least 90 days

^4^At least one check-up by health professionals within 2 weeks of delivery

^5^Referred to as institutional delivery conducted by SBA in this study

^6^ANC visit in the first trimester and > 4 ANC visits

^7^> 3 ANC, at least two TT, and had received IFA

^8^Within 2 days after delivery

^9^Within 42 days after delivery

^10^Referred to as using health facility for delivery in this study

^11^Enabled Action Group

## Abbreviations

ANC: Antenatal care
ASHA: Accredited Social Health Activist
ANM: Auxiliary nurse-midwives
CSDH: The Commission on Social Determinants of Health
JSY: Janani Suraksha Yojana
MMR: Maternal mortality ratio
NFHS: National Family Health Survey
OBC: Other backward caste
OR: Odds ratio
PHC: Primary healthcare center
PNC: Postnatal care
SC: Scheduled caste
SEBC: Socially and economically backward caste
ST: Scheduled tribe
TT: Tetanus toxoid
UNDP: United Nations Development Programme

## Footnotes

1 JSY, or literally translated Mother Protection Scheme, promotes facility-based deliveries through cash incentives for pregnant women and Accredited Social Health Activists (ASHAs).

2 ASHAs are female community health workers who would be the first point of call for any health problem in a village and would link communities with health facilities [77].
